# BiCluE - Exact and heuristic algorithms for weighted bi-cluster editing of biomedical data

**DOI:** 10.1186/1753-6561-7-S7-S9

**Published:** 2013-12-20

**Authors:** Peng Sun, Jiong Guo, Jan Baumbach

**Affiliations:** 1 Computational Systems Biology Group, Max Planck Institute for Informatics, Campus E1.4, 66123 Saarbrücken, Germany; 2 Cluster of Excellence for Multimodal Computing and Interaction, Saarland University, Campus E1.7, 66123. Saarbrücken, Germany

## Abstract

**Background:**

The explosion of biological data has dramatically reformed today's biology research. The biggest challenge to biologists and bioinformaticians is the integration and analysis of large quantity of data to provide meaningful insights. One major problem is the combined analysis of data from different types. Bi-cluster editing, as a special case of clustering, which partitions two different types of data simultaneously, might be used for several biomedical scenarios. However, the underlying algorithmic problem is NP-hard.

**Results:**

Here we contribute with BiCluE, a software package designed to solve the weighted bi-cluster editing problem. It implements (1) an exact algorithm based on fixed-parameter tractability and (2) a polynomial-time greedy heuristics based on solving the hardest part, edge deletions, first. We evaluated its performance on artificial graphs. Afterwards we exemplarily applied our implementation on real world biomedical data, GWAS data in this case. BiCluE generally works on any kind of data types that can be modeled as (weighted or unweighted) bipartite graphs.

**Conclusions:**

To our knowledge, this is the first software package solving the weighted bi-cluster editing problem. BiCluE as well as the supplementary results are available online at http://biclue.mpi-inf.mpg.de.

## Introduction

### Background

**Figure 1:**
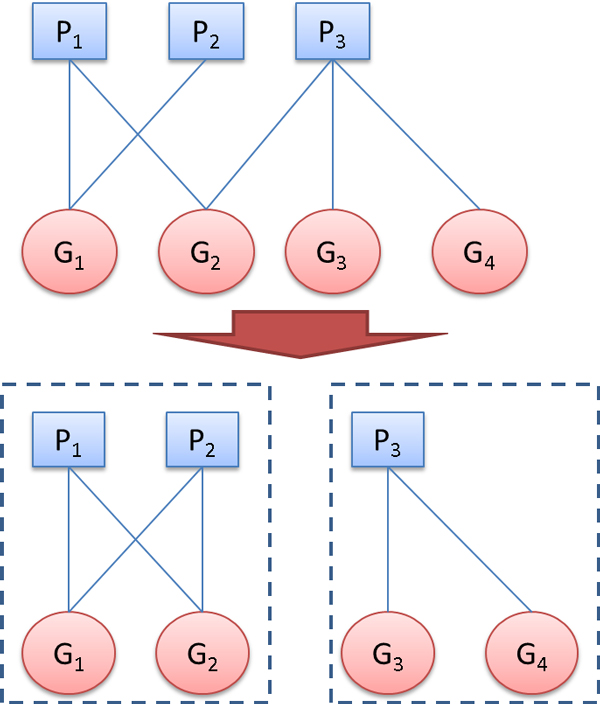
**Bipartite graph representation of GWAS data**. Vertices *P*_1_, *P*_2_, *P*_3 _represent "phenotype" and *G*_1_, *G*_2_, *G*_3_, *G*_4 _refer to "genotype" (SNPs). Our BiCluE approach converts the intransitive GWAS data graph into disjoint bi-cliques. One possible solution is presented at the bottom: The insertion of edge *P*_2 _and *G*_2 _and the deletion of the edge between *P*_3 _and *G*_2_.

The enormous amount of available (sequential) data from laboratories around the world has greatly shifted the focus of biologically motivated studies. For instance, GenBank, as the largest database of genes, now stores over 197,000,000 sequences of more than 380,000 organisms [1]. UniProtKb/Swiss-Prot provides a database containing more than 53,000 annotated sequences, extracted and integrated from 205,244 published references and Protein Data Bank (PDB) has incorporated over 78,400 molecule structures. Integrating, processing and analyzing large quantities of data from various sources have become the main challenge in modern bioinformatics. The requirement of carefully designed computational models and methodology increases rapidly, in order to discover novel interrelations and gain further insights. In our study, we focus on the exact and heuristic algorithms that cluster data from different types simultaneously, i.e. so called "bi- cluster editing". A software package named BiCluE containing an exact algorithm and a heuristic algorithm is available for downloading http://biclue.mpi-inf.mpg.de. We test and evaluate BiCluE on artificially generated data. Afterwards, we demonstrate its applicability to real-world Genome-Wide Association Study data, also known as GWAS. GWAS examines the genetic variants (genotypes) of one species, aiming for searching associations with a certain phenotypic trait. In one typical GWAS project, millions of SNPs are investigated and statistical tests are performed to verify significant associations with the phenotypes. This is a typical example of a bipartite data type, i.e. two types of data objects and a measurement that finds relations between the instances of two types. Traditional analysis of GWAS data associates only one pair of SNP and trait/disease in one statistical test. This methodology tends to have false positives and false negatives and to overlook the joint effects of moderate risk SNPs. Therefore, we changed this strategy, forming "group to group" associations, rather than the traditional "one to one" relations (Figure 1). In our previous study [2], a preliminary version of exact algorithm was implemented. Now we bring forward a newly designed heuristics, largely shortening the running time without significant compromising the accuracy. A software package was implemented to integrate both two algorithms and make it more convenient for people to use. Both two algorithms were applied to the GWAS data set and were capable of dealing with all of the 415 problem instances but two, which have been too large (> 3,500 nodes). 86 putative new associations were discovered and we hope our results can serve as a guide for further investigations in the wet lab. In other words, we seek to explain the joint effect of multiple genotypes to multiple phenotypes by "virtually" adding/removing associations such that bi-cliques emerge in the underlying bipartite graph. Note that we chose GWAS data as an intuitive real-world example for data that can be modeled as bipartite graphs. Since BiCluE can be applied to many different biomedical data types (see Results section), the focus of this paper lies on the exact fixed-parameter algorithm for weighted bi-cluster editing and on the edge deletion heuristics.

### Cluster editing and bi-cluster editing

Clustering is a classical task in bioinformatics and computational biology. It partitions a data set into different clusters such that elements within a cluster are more similar to each other than to those objects belonging to different clusters, according to a certain criterion. Various clustering methods are used in every field of biological studies, including functional genomics, protein structure/sequence analyses and almost all types of network analyses (e.g., transcription regulatory network, protein-protein interaction networks) [3]. Some specific types of clustering were designed for different scenarios. For instance, the clustering of gene expression data under different conditions, which can be modeled as a bipartite graph [4], is hardly suitable for standard clustering methods. Instead, one would like to cluster genes and conditions simultaneously such that we see a consistent "behavior", i.e. so called bi-clustering.

Clustering and bi-clustering are very similar problems, thereby sharing similar strategies. One of the common approaches of solving the problems is to compute a pair-wise similarity matrix and to choose a similarity threshold for constructing the corresponding similarity graphs. Such graphs are built according to the following steps: (1) The vertices of the graph refer to the objects (for instance, genes or conditions), and (2) an edge between two objects is drawn if the similarity score between two vertices is above a certain threshold [5]. We call two arbitrary vertices *u *and *v *"similar" when the score is above a certain value, written as *u ~ v*. However, the resulting graph is not necessarily transitive, meaning for arbitrary three vertices *uvw*, *u ~ v *and *u ~ w *does not necessarily imply *v ~ w*. As a result, we aim to convert the preliminarily constructed graph into a transitive graph, which is a disjoint union of cliques, with minimal costs (minimal number of edge deletions/insertions, for instance). Such a problem is named "cluster editing". The formal problem statement follows:

Let *V *be the set of vertices (objects) to be clustered and *uv *be an unordered pair of elements in *V *, i.e., $\left\{u,\;v\right\}\in \left(\begin{array}{c} V\\ 2 \end{array}\right)$. We then define the similarity between two vertices as a symmetric function *s *: $\left(\begin{array}{c} V\\ 2 \end{array}\right)$ → *R*. A given threshold is then used to decide whether *u *and *v *are "similar " (if *s*(*uv*) *≥ threshold*) or "not similar" (if *s*(*uv*) <*threshold*). Let *E *= {*uv *: *u ~ v*} denote the edge set of the similarity graph. Here in this study, self-loops are not permitted.

The graph is called transitive, if it satisfies any of the three equivalent conditions below:

• Every set of three vertices $uvw\;\;\in \;\;\left(\begin{array}{c} V\\ 3 \end{array}\right)$ satisfies: *uv *∈ *E *and *vw *∈ *E ⇒ uw *∈ *E*.

• No path of three vertices is allowed.

• Every disjoint component of *G *is is a clique. (A clique is a complete graph.)

Given an input graph *G *= (*V, E*), one asks whether *G *can be transformed into a transitive graph $G^{\prime }=\;\left(V,\;E^{\prime }\right),$ by inserting and deleting edges. For each insertion or deletion, we have a certain penalty depending on *s*(*uv*). Let $cost\left(G\;\to G^{\prime }\right)=s\left(E\backslash E^{\prime }\right)-s\left(E^{\prime }\backslash E\right)$ denote the cost function. Our task is to find a $G^{\prime }$, such that $cost\left(G\;\to G^{\prime }\right)$is minimized.

Bi-cluster editing, similar to "cluster editing", is a mathematical model of the "bi-clustering" problems, also serving as a strategy of solving bi-clustering problems. The graphs are built in the same way, with vertices referring to the entities and edges representing similarities. However, the resulting graph must be a bipartite graph. Bipartite graphs are special graphs satisfying the following criteria: (1) the vertices of the graph are divided into two subsets, and (2) edges can only be defined between vertices belonging to different subsets.

We consider a bipartite graph *G *= (*V, E*) transitive if it satisfies any of the following equivalent conditions:

• For an arbitrary subset of four vertices, $uvwx\;\;\in \;\;\left(\begin{array}{c} V\\ 4 \end{array}\right)$, where *u, w *belong to the same subset and *v*, *x *belong to the other, we have *uv *∈ *E *, *wv *∈ *E *and *wx *∈ *E ⇒ ux *∈ *E*.

• No paths of 4 vertices can be found, i.e., for each $uvwx\in \;\;\left(\begin{array}{c} V\\ 4 \end{array}\right)$, where *u, w *belong to the same subset and *v, x *belong to the other, we have |*E *∩ {*uv, wv, ux, wx*}| ≠ 3.

• *G *is a union of disjoint bi-cliques (i.e. complete bipartite graphs).

Bi-cluster editing is similar to its counterpart, cluster editing: We transform a given bipartite graph into a union of disjoint bi-cliques by edge insertions and deletions with minimal costs for these modifications. The definition of $cost\left(G\;\to G^{\prime }\right)$ is the same. Note that bi-cluster editing, though related to biclustering (see [6]), is different in concept, methodology and biomedical applicability.

### Problem statement

The weighted bi-cluster editing problem is defined as follows: Given an undirected bipartite graph *G *= (*V, E, s*), where *s *is a similarity function $s:\;\left(\begin{array}{c} V\\ 2 \end{array}\right)\to R$. Let $G^{\prime }$ be a union of disjointed bi-cliques. Find one or all $G^{\prime }$ such that $cost\left(G\;\to G^{\prime }\right)$ is minimized.

The input to our algorithm is a graph *G *= (*V, E, s*), with similarity function *s*(*uv*) *→ R *and a similarity threshold. *E *denotes the set of the edges: *E *= {*v*_1_*, v*_2 _: *s*(*v*_1_*v*_2_) *> threshold*}. The algorithm outputs a set of edited edges E* and a cost c* = *cost*(*G → *(V, E\E* ∪ E*\E).

We assume that the input graph consists of only one single connected component since we can apply the algorithms on each connected component separately, without loss of generality. An optimal solution of the bi-cluster editing problem would never join separate components, since we can always find a cheaper solution where all separated components remain separated [7].

In this study, we use "P4" as the short from of "a path of 4 vertices". As mentioned above, a bipartite graph is transitive if and only if it contains no P4. Denote *B*(*G*) to be the set of all P4s, i.e. $B\left(G\right)\;=\left\{uvwx\;\;\in \;\;\left(\begin{array}{c} V\\ 4 \end{array}\right)\left|\;\right|E\;\cap \left(uv,\;wv,\;ux,\;wx\right)|\;=\text{ 3}\right\}$. *G *is transitive if and only if *B*(*G*) = ∅.

### Previous studies and results

It has been proven that both unweighted and weighted bi-cluster editing problems are NP-hard [8]. Although many studies focused on cluster editing [3,5,9], the study of bipartite transitive graph projection is far from complete. An algorithm based on graph module decomposition for unweighted bi-cluster editing was developed by F. Protti *et al*., with the time complexity of *O*(4*^k ^*+ |*V*| + |*E*|) [10]. Later J. Guo *et al*. improved the running time to *O*(3.24*^k ^*+ |*E*|), by a refined branching strategy [7]. However, we still lack algorithms solving weighted bi-cluster editing problem instances, which covers most of the cases in real life.

### Fixed-parameter algorithm

Fixed-parameter algorithm and fixed-parameter tractability were first introduced by Downey and Fellows in 1990s as a methodology of solving NP-hard problems more efficiently [11]. An NP-hard problem is called "fixed-parameter tractable", if it can be determined with a running time complexity that increases polynomially with input size and exponentially or worse with the parameter *k*, namely *O*(*f*(*k*)·|*I*|^*c*^), where |*I*| is the input size and *c *is a constant. Moreover, *f *must be a function that only depends on *k*. Niedermeier gives a more detailed introduction to the theories and applications of fixed-parameter algorithms and fixed-parameter tractability [12].

### Our contributions

Here, we present BiCluE, a Java software package that deals with weighted bi-cluster editing. BiCluE implements an exact algorithm based on fixed-parameter tractability theory and a new faster running heuristic algorithm based on optimal edge deletion estimation. We regard the parameter *k *of the fixed-parameter algorithm as the cost of edge modifications. Given a problem instance, the exact algorithm finds the optimal solution with cost at most *k*, if there is such a solution. Assuming |*s*(*uv*)|>1 for all possible *u, v*, our exact algorithm finishes in *O*(4*^k^*) time, checking whether there is an optimal solution or not, while the heuristic algorithm needs *O*(|*E*|·(|*E*| + |*V*|^2^) + |*V*|^3^) time to output an "approximate" optimal solution.

Although, we focus on the new algorithms here, we evaluated our BiCluE approach on artificially generated graphs. Afterwards, we exemplarily show that BiCluE may be applied to real biomedical data by means of two different GWAS data sets. In this intuitive setting of a bipartite graph we used our weighted bi-cluster editing algorithms to scan for new putative associations that can be deduced from the resulting "group to group" relations. We will discuss the new findings and hope that our results can serve as guidelines for further statistical investigations and wet lab studies.

## Methods

### Fixed-parameter algorithm

Our fixed-parameter algorithm contains two important parts: Data Reduction and Branching Strategy.

Data Reduction: The data reduction is a kind of preprocessing of the fixed-parameter algorithm that reduces the instance size by removing those parts of the problem instance that do not need to be repaired and thereby do not need to be considered in the following steps. We first recognize all the separate components as individual input. Then the algorithm checks whether each component is already a bi-clique or not. If this is the case, then the algorithm removes the whole component from the input and outputs it as a part of the solution. This procedure finishes within *O*(|*V*| + |*E*|) time.

**Figure 2:**
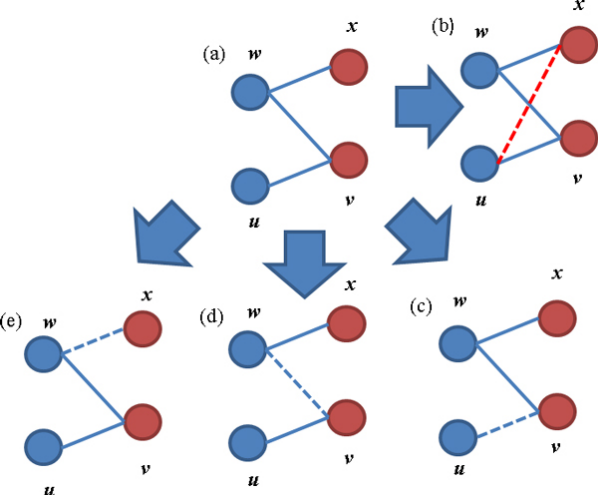
**The bi-cluster editing strategy based on P4-branching**. Blue dashed lines correspond to edge deletions and red dashed lines correspond to edge insertions. Four possibilities of repairing a P4 are presented: (b) Insertion of the missing edge *ux*; (c) deletion of the edge *uv*; (d) deletion of the edge *wv*; and (e) deletion of the edge *wx*.

Branching Strategy: Branching strategy refers to a search tree procedure to search and edit the P4s using edge insertions and deletions. We have 4 possibilities to convert a P4 into bi-cliques: removing one of the three edges, resulting in two bi-cliques, or complete the P4 with one edge insertion (Figure 2). More specifically:

Suppose *uvwx *is an arbitrary P4 and let (*uv*), (*wv*), (*wx*) be the three edges in the P4. The following three cases are checked recursively as shown in Figure 2a:

• Insert *ux *by setting the weight of *ux *to "permanent" (Figure 2b)

• Delete *uv *by setting the weight of *uv *to "forbidden" (Figure 2c)

• Delete *wv *by setting the weight of *wv *to "forbidden" (Figure 2d)

• Delete *wx *by setting the weight of *wx *to "forbidden" (Figure 2e)

The search tree procedure starts when a P4 is located. Four branches are created for one P4 in the search tree; each represents one of the editing possibilities. Then we recursively visit the four branches one by one, performing the corresponding edge insertions or deletions and update *k *to $k^{\prime }$ = *k−*(insertion or deletion cost). We implement the whole algorithm in a recursive manner. If the editing behavior of a certain branch leads to $k^{\prime }\;<\;0$, then the corresponding branch is skipped. The algorithm stops when the entire tree is visited, and returns the optimal solution found. This branching strategy accepts a worst case running time of *O*(4*^k ^*).

### Edge deletion heuristics

In the fixed-parameter algorithm, we are aiming for repairing all the P4s to make *G *transitive. The repairing behavior is either an edge insertion or edge deletion. It is obvious that the difficult part of the problem is to correctly locate the edge deletions, for the edge deletions determine the number of resulting disjoint bi-cliques. Therefore, it would be beneficial to find the most promising positions of edge deletions first. Then edge insertions can easily be carried out by inserting all the edges required to make each disjoint component transitive. This is the main idea behind our edge deletion heuristic algorithm.

We define a function to score the edge removal candidates and greedily delete the edge with highest score in each step, until further deletions do not improve the solution. For each P4 *uvwx *(where (*uv*), (*wv*), (*wx*) ∈ *E*), we define deviation from transitivity of *G, D*(*G*) as:

$$D\left(G\right)\;=\;\underset{uvwx\in P\text{4}}{\sum }\text{min}\left\{\left|s\left(uv\right)\left|,\;\right|s\left(vw\right)\left|,\;\right|s\left(wx\right)\left|,\;\right|s\left(xu\right)\right|\right\}$$

The score of edge deletions are computed as follows: Let *uv *be an arbitrary edge in *G *= (*V, E, s*). $G^{\prime }\;=\;\left(V,\;E\backslash \left\{uv\right\},\;s^{\prime }\right\}$ is *G *after the removal of *uv*, where $s^{\prime }\left(xy\right)\;=\;s\left(xy\right)$, except $s^{\prime }\left(uv\right)\;=\;-\infty$ (*uv *set to "forbidden"). Then we define:

$$\Delta_{uv}\left(G\right)\;=\;D\left(G\right)\;-\;D\left(G^{\prime }\right)\;-\;s\left(uv\right)$$

as the *transitivity improvement *of edge *uv *where *s*(*uv*) is the cost of edge deletion.

The edge deletion heuristic algorithm consists of three functions: REMOVE_CULPRIT(*G*), TRANSITIVE_CLOSURE_COST(*G*) and EDGE_DEL_MAIN(*G*). REMOVE_CULPRIT(*G*) returns the edge with highest *transitivity improvement *(*argmax_uv∈E_*{Δ*uv*(*G*)}) and removes it from *G*; TRANSITIVE_CLOSURE_COST(*G*) returns the total cost of all edge insertions required to convert *G *into a bi-clique, assuming *G *is connected; EDGE_DEL_MAIN(*G*) is the main function of the edge deletion heuristic.

The first invocation of REMOVE_CULPRIT(*G*) can be finished in *O*(|*E*|·|*V*|^2^) time, since computing each Δ_*uv*_(*G*) can be finished in *O*(|*V*|^2^) time, for only those P4s that contain *uv *are considered. The subsequent routine calls require *O*(|*V*|^2^) time to update the scores of the edges that were influenced by the deletion of *uv*, and finally *O*(|*E*|) time to find the maximum scored edge. This results in a total running time of *O*(|*V*|^2 ^+ |*E*|). TRANSITIVE_CLOSURE_COST(*G*) sums up the cost for a transitive closure, accepting a running time of *O*(|*V*|^2^).

EDGE_DEL_MAIN(*G*) returns a solution object, containing the edge modifications and the costs needed for converting the input graph into a transitive one. We keep the assumption that *G *is connected. The pseudo-code of the EDGE_DEL_MAIN(*G*) is in the appendix.

Our edge deletion heuristics removes a specific edge at most once across all recursions. Checking for connected components requires *O*(|*E*| + |*V*|) time and REMOVE_CULPRIT(*G*) requires *O*(|*V*|^2 ^+ |*E*|) time. TRANSITIVE_CLOSURE_COST(*G*) takes *O*(|*V*|^2^) time for each disjoint component. Therefore, the total running time of our algorithm is *O*(|*E*|(|*E*| + |*V*|^2^) + |*V*|^3^).

## Results

### Artificial graphs

The artificial graphs were generated as follows: Initially we generate graphs consisting of *n *vertices; afterwards *m *vertices are picked up (*m *∈ [1, *n*]) and defined to be in one bi-clique. Then the same procedure is carried out in the remaining *n−m *vertices until there is no vertex left. This random graph generating process gives us a graph consisting of random numbers of clusters of random sizes. The edge weights are obtained from two different Gaussian distributions *N *(*μ_intra_*, $\sigma_{intra}^{2}$) and *N *(*μ_inter_*, $\sigma_{inter}^{2}$). The former distribution is to generate weights for edges within the pre-designed bi-clusters and the latter for "inter bi-cluster edges", i.e. the edges between vertices connecting different bi-clusters. We chose *μ *and *σ *carefully such that the generated graphs are "almost transitive" bipartite graphs. In our case, *μ_intra _*= 21*, μ_inter _*= *−*21*, σ_intra _*= *σ_inter _*= 18. Thus the probability of finding a "mistake edge" (an edge between vertices in different bi-clusters or a missing edge between vertices in the same bi-cluster) is about 0.123 for each pair.

**Figure 3:**
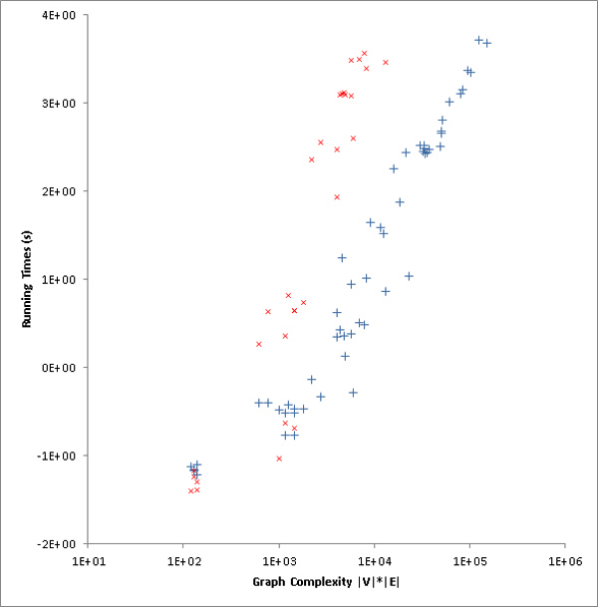
**Running times of fixed-parameter algorithm and edge deletion heuristics against graph complexities**. The figure shows the running times against graph complexities, i.e. |*V*|·|*E*| for artificial graphs. Note the log-scaling of the axes. Colors: blue - edge deletion heuristics, red - fixed-parameter algorithm.

The performance of the two algorithms on artificial graphs are shown in Table 1. All running time measurements were averaged over 5 repeats of the same-sized graphs but with different edge structures. The fixed-parameter algorithm is able to achieve very small running times on small-sized and medium-sized graphs, yet as the sizes of graphs grow, the performance of fixed-parameter algorithm suffer, indicating the NP-hardness of the underlying problem. When the number of vertices exceeds 40 vertices, the fixed-parameter algorithm cannot finish within reasonable time. On the other hand, the edge deletion heuristic algorithm requires significantly less time than the fixed-parameter algorithm on bigger graphs. In terms of costs, the performance of edge deletion heuristics is almost as good as that of the fixed-parameter algorithm. In summary, the heuristic finds solutions that are almost equally good but is significantly faster. In Figure 3 we plot the running times of the fixed-parameter algorithm and the edge deletion heuristic against the graph component complexity (we define graph complexity as |*V*|·|*E*|). Obviously, fixed-parameter is faster for small components, but running times explode with growing input graphs sizes. The edge deletion heuristics performs better in terms of running time for medium-sized components without much worse accuracy, i.e. much higher modification costs.

**Table 1:** Results of BiCluE on artificial graphs with a varying numbers of vertices.

No. of Vertices	No. of Edges	Costs		Running Times(s)	
		
		**FP**.	**EDH**.	**FP**.	**EDH**.
10	[12,14]	**23.786**	25.575	**0.0514**	0.0704

20	[31,73]	**114.024**	114.934	**0.0940**	0.320

25	[42,101]	**106.902**	116.36	5.061	**0.318**

30	[74,190]	**197.510**	209.9	275.336	**1.626**

35	[126,170]	**339.941**	345.232	744.231	**5.212**

40	[144.326]	**437.44**	454.713	2183.653	**6.578**

50	[185,468]	(--)	652.204	(--)	40.601

60	[266,635]	(--)	943.902	(--)	260.957

70	[430,740]	(--)	1397.101	(--)	376.010

80	[632,1341]	(--)	1784.506	(--)	711.245

90	[936,1679]	(--)	2243.770	(--)	1670.032

Costs and running times are averaged over 5 runs. For graphs with ≤ 40 vertices, we applied both algorithms. For graphs with > 40 vertices, the fixed-parameter algorithm cannot finish within reasonable time. Hence, we only report the performance of the edge deletion heuristics. Better costs and running times are marked with bold font. Abbreviations: FP: fixed-parameter algorithm; EDH: edge deletion heuristics

**Figure 4:**
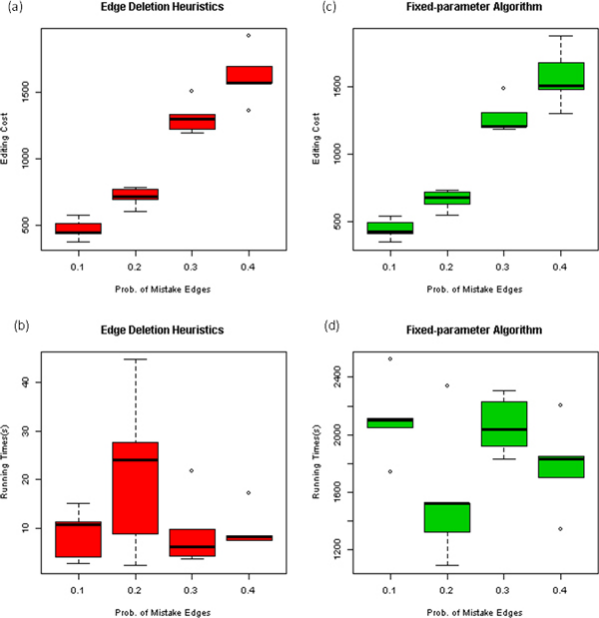
**The variation of costs and running times of our two algorithms on randomly generated input graphs with varying "mistake edge probabilities"**. (a),(b): The costs and running times for edge deletion heuristics. (c), (d): The costs and running times for fixed-parameter algorithm.

We also generated 20 random graphs for different probabilities of "inter-edges" and "intra-missing-edges" (see above) ranging from 0.1 to 0.4 (5 repeats for each probability). The average graph size was |*V*| = 40. The solutions have been computed within one hour for all graphs. The box plots in Figure 4 show the variation of running time and costs for these graphs. With higher "mistake probabilities", the costs are expected to be higher as well. For run times, however, we do not see an increasing trend. Hence, our methods seem to be generally robust for graphs of different structures and complexities. The results are shown in Figure 4.

### Genome wide association studies

To demonstrate the applicability of BiCluE to real biomedical data, we applied our software to a very intuitive bipartite graph data set: GWAS data. It was retrieved from two sources: (1) an online available database [13], containing 56,412 significant SNP associations for 87 different diseases/traits, and (2) the National Human Genome Research Institute (NHGRI) Catalog of Published Genome Wide Association Studies, an online catalog of SNP-traits from published GWASs, with 5,476 unique SNPs and 526 different diseases [14]. We defined edge weights as *s*(*uv*) = *−log*(*P*), where *P *is the *p*-value of the given associations. We used a threshold of 0.05, corresponding to -*log*(0.05) = 1.301 in our graph-based model.

The data from two different databases were not merged due to incompatibility of terminology. The resulting graphs contain 415 connected components in total (136 from Johnson's dataset and 279 from the NHGRI dataset).

Both fixed-parameter algorithm and edge deletion heuristics were applied separately on each disjoint connected component. 413 of 415 components were solved within 24 hours (99.5% of all the components). The remaining two graph components have been too big to be solved within 24 hours (one from NHGRI dataset with |*V*| = 3, 609 and one from Johnson's dataset with |*V*| = 50, 161). Both BiCluE algorithms identified 86 putative associations, which were not detected as significant in the two GWAS datasets. Table 2 shows the distribution of the new associations and their corresponding diseases/traits. We found 11 new targets to be tested and evaluated for "Conduct disorder (case status)" and "Isochemic Stroke", followed by "Atrial fibrillation/atrial flutter" and "Permanent tooth development" (10 new candidate associations). For the details of the putative associations, please refer to Additional File 1. Note that our predictions depend on the user-given similarity threshold, as those of any other clustering tool. A full analysis of all the putative association is beyond the scope of this study. Nevertheless, we examined the previous reports and literature for further supports. The SNP rs2548145 and rs3930234, which were discovered in our study as putatively associated to "Alcoholism (alcohol dependence factor score)" have been reported as associated to "Alcoholism (alcohol use disorder factor score)" [15]. Moreover, the SNP rs13376333, which was studied and found associated with "Atrial fibrillation" [16], was found to be putatively associated to "Atrial Flutter" and "Ischemic stroke". Another interesting result is related to the trait of "tooth development": SNP rs9674544 and rs1956529, reported as related to "primary tooth development" [17], were found to be associated with "permanent tooth development" in our study. Although the analyses above could not replace experiment verifications as the "ultimate" validation, yet it demonstrates that BiCluE tends to cluster related traits together in one group, which further implicates the correctness of the putative associations. However, final wet lab examination is still necessary and indispensable, though beyond the scope of this paper. Here, our focus is the new BiCluE algorithms that solve the weighted bipartite graph cluster editing problem. As any other data partitioning method it needs further statistical testing and parameter adjustment, which is highly application-specific and needs to be done with a certain intuition regarding the nature of the real-world data sets.

**Table 2:** Putative associations obtained from bi-cluster editing.

Traits/Disease	No. of Putative Associations
Conduct disorder (case status) *	11

Ischemic stroke	11

Atrial fibrillation/atrial flutter*	10

Permanent tooth development*	10

Conduct disorder (symptom count)*	9

Primary tooth development (time to first tooth eruption)*	8

Cleft lip*	7

Primary tooth development (number of teeth)*	5

Alcoholism (alcohol dependence factor score)*	4

Plasma coagulation factors*	3

Vitamin D insufficiency*	3

Vitamin D levels*	2

Atrial fibrillation*	1

Nonsyndromic cleft lip with or without cleft palate*	1

Plasma levels of Protein C*	1

Total	86

The items with "*" come from NHGRI dataset and the rest are from Johnson *et al*.'s online dataset.

## Discussion and conclusion

Here, we presented BiCluE, a software package dedicated to solve (weighted) bi-cluster editing problems. It offers a fixed-parameter algorithm and an edge deletion heuristic. We showed that BiCluE is able to solve medium-sized bi-cluster editing problems within reasonable times. The running times of fixed-parameter algorithm explode when the input size exceeds a certain value (40 vertices) while the edge deletion heuristic still works fine for graphs of larger sizes.

We demonstrated BiCluE's ability to cluster biomedical data with publicly available GWAS data sets. All but two instances (99.5%) have been solved. We found 86 putative new associations. These newly discovered associations might be useful as guidelines for further wet lab studies. Since deleting/inserting edges (associations between a phenotype and a SNP) does not directly affect the association of other SNPs to that phenotype, we implicitly imply a certain degree of independence between SNPs, which might not be true. However, when a set of SNPs is highly connected to a set of phenotypes, it is likely that we may neglect inter-SNP dependencies, since we concentrate on inserted edges (Table 2) emerging from the "group-to-group" relationship in the bipartite graph.

Moreover, there are plenty of other potential applications of BiCluE. In the future, we will apply BiCluE to identify genetic variants that are responsible for certain bacterial life styles, a task that will require simultaneous clustering of both, genes and species. We will investigate more such applications in the future.

Further investigation will focus on the improvement of the performance of the fixed parameter algorithm. The counterpart of bi-cluster editing on general graphs, cluster editing, has been extensively studied. Thus it might be interesting to compare the two problems, making use of the ideas and techniques for cluster editing problems on bi-cluster editing in order to achieve better running times.

## Implementation

BiCluE is implemented in JAVA 1.6 with support for parallel multi-core computing. All measurements for the evaluations were taken on Compute Clusters with 78 computing nodes consisting of 2 × Intel XEON E5430 2.66 Ghz (Quad-core) CPUs and 16 GB RAM.

## Competing interests

The authors declare that they have no competing interests.

## Authors' contributions

PS designed and implemented the algorithm. JG and JB supervised the whole work. All authors contributed equally to the manuscript.

## Supplementary Material

Additional file 1 **Null**Null Click here for file
