# Supplementary material for: BiCluE - Exact and heuristic algorithms for weighted bi-cluster editing of biomedical data
Source: BMC Proc. 2013 Dec 20;7(Suppl 7):S9. doi: 10.1186/1753-6561-7-S7-S9 (PMC4044484; doi:10.1186/1753-6561-7-S7-S9)
Supplement: Additional file 1 — NullNull [file 1753-6561-7-S7-S9-S1.PDF]

Additional Files

Algorithm - the pseudo code for edge deletion heuristics

Algorithm 1 EDGE_DEL_MAIN(G)

```
cost = cost ←TRANSITIVE_CLOSURE_COST(G);
if (cost == 0) then
    return(null, 0);
end if
actions ← null; delcost ← 0;
while (G is still connected) do
    uv ← REMOVE_CULPRIT(G);
    actions.add(uv);
    delcost+ = s(uv);
end while
// Adjust actions such that it only contains the edge removals contributing to the separation of two
subgraphs of G
//Assume G is cut into G1 and G2
while (uv in actions) do
    if (both u, v are in G1 or G2) then
        actions.remove(uv);
    end if
end while
//Solve the problem in a recursive manner for G1 and G2, until no better solution can be found
if (delcost >= cost) then
    return(null, cost);
end if
(list1, cost1) ← EDGE_DEL_HEURISTICS(G1);
if (delcost + cost1 >= cost) then
    return(null, cost);
    (list2, cost2) ← EDGE_DEL_HEURISTICS(G2);
end if
if (delcost + cost1 + cost2 >= cost) then
    return(null, cost);
end if
actions.add(list1);
actions.add(list2);
//add all the edge insertions required for the closure of transitivity
actions.add(insertions);
return(actions, delcost + cost1 + cost2);
```

Additional Table 1 - The details of the 86 putative GWAS associations
